# Lipid Lowering Effects of Hydroalcoholic Extract of *Anethum graveolens* L. and Dill Tablet in High Cholesterol Fed Hamsters

**DOI:** 10.1155/2015/958560

**Published:** 2015-12-28

**Authors:** Ebrahim Abbasi Oshaghi, Iraj Khodadadi, Massoud Saidijam, Reza Yadegarazari, Nooshin Shabab, Heidar Tavilani, Mohamad Taghi Goodarzi

**Affiliations:** ^1^Department of Clinical Biochemistry, School of Medicine, Hamadan University of Medical Sciences, Hamadan 6517838736, Iran; ^2^Research Center for Molecular Medicine, Hamadan University of Medical Sciences, Hamadan 6517838736, Iran

## Abstract

*Objective*. This study was aimed to determine the effect of* Anethum graveolens* extract and* Anethum graveolens* (dill) tablet on lipid profile, liver enzymes, and gene expression and enzymatic activity of HMG-CoA reductase in high cholesterol fed hamsters.* Materials and Methods*. Golden Syrian male hamsters (130 ± 10 g) were randomly divided into 6 groups (*n* = 6) and received daily the following: group 1 received chow + 2% cholesterol + 0.5% cholic acid (HCD), groups 2 and 3 received HCD diet plus 100 and 200 mg/kg hydroalcoholic extract of dill, respectively, and groups 4 and 5 received HCD diet plus 100 and 200 mg/kg dill tablet, respectively. Group 6 received only chow. After 1 month feeding serum biochemical factors were determined. HMG-CoA reductase mRNA level was measured (real-time PCR) and its activity was determined spectrophotometrically.* Results*. Compared with hypercholesterolemic group 1, lipid profile, blood glucose, and liver enzymes significantly decreased in all dill tablet or dill extract treated groups (*p* < 0.05). The changes in HMG-CoA reductase gene expression level and enzyme activity significantly reduced in animals that received 200 mg/kg of extract or tablet.* Conclusion*. Dill extract and dill tablet showed potential hypocholesterolemic properties in hamsters by inhibition of HMG-CoA reductase activity.

## 1. Introduction

Hypercholesterolemia is a common disorder which is known as main cause of coronary heart disease (CHD) [1]. This disease is recognized as cause of the most of the deaths in developed countries. High levels of total cholesterol (TC), triglycerides (TG), and low density lipoprotein-cholesterol (LDL-C) have been implicated as contributing risk factors in progress of CHD and atherosclerosis [2]. Accordingly, different lipid lowering drugs have been applied for the treatment of hypercholesterolemia [3]. Statins are the effective hypocholesterolemic drugs which competitively inhibit the activity of 3-hydroxy-3-methylglutaryl-coenzyme A (HMG-CoA) reductase, the rate limiting enzyme in cholesterol biosynthesis [4]. Since chemical drugs, for example, statins, have adverse effects such as rhabdomyolysis, myopathy, myalgia, and myotoxicity [4], applicability of herbal medicine for treatment of many diseases has attracted much more attention. Some herbal medicines have been known to exhibit hypocholesterolemic properties in clinical studies [5]. One of the useful and well-known plants is* Anethum graveolens* L., commonly known as a dill, an annual plant growing in Europe, Mediterranean region, and Asia [6]. In traditional medicine, this plant has been used for the treatment of gastrointestinal disorder including indigestion, flatulence, and stomacha colic and also for its antibacterial, antifungal, antispasmodic, antisecretory, mucosal protective, and hypoglycemic effects. Dill tablet (DT) which is administrated as lipid lowering agent contains* Citrus aurantifolia* sp. (4%),* Cichorium intybus* (5%),* Fumaria parviflora* (5%), and* Anethum graveolens* (68%) [6]. Previous studies have reported the presence of phenolic and flavonoids, flavonol, alkaloids, anthocyanin, tannin, and saponin contents in dill [6]. Studies have established that phenolic and flavonoids have potential antioxidant and hypocholesterolemic effects [7]. The hypocholesterolemic activity of dill has been shown in different studies, but the lipid lowering mechanism has remained unknown so far. Therefore, the aim of this study was to investigate the hypocholesterolemic effects of the dill in high cholesterol fed hamsters and to determine gene expression level and enzymatic activity of HMG-CoA reductase in liver.

## 2. Materials and Methods

### 2.1. Preparation of Dill Extract

Dill was purchased from Hamadan (west of Iran) daily market and identified by our colleagues in the Department of Biology, Borujerd Azad University (Borujerd, Iran). Hydroalcoholic extract was prepared according to the previously described method [5]. Dill tablet (DT) was purchased from Iran Darouk Company (Tehran, Iran).

### 2.2. Determination of Total Phenolic Content

The amount of total phenolic content of dill extract and/or dill tablet was determined according to previously reported method with small modification using Folin-Ciocalteu reaction [6]. Briefly, 1 mg of dill was dissolved in 3.8 mL of deionized water, 2 mL of Na_2_CO_3_ (2%), and 0.1 mL of Folin-Ciocalteu reagent (50%). The samples were then incubated for 30 min at room temperature and the absorbance of the reaction mixture was measured at 750 nm against deionized water. Total phenolic content was calculated per mg equivalents of gallic acid (GAE) per gram of each extract.

### 2.3. Determination of Total Flavonoids

Total flavonoids were measured using aluminum chloride colorimetric assay, according to the previously described method [6]. Briefly 0.5 mL of the sample (1.0 mg/mL in methanol) was mixed with 1.5 mL of alcohol (95%), 0.1 mL of AlCl_3_ (10%), 0.1 mL of potassium acetate (1 M), and 2.8 mL of deionized water. After that, the mixture was incubated at room temperature for 40 min and absorbance of the mixture was measured at 415 nm against deionized water. The content of flavonoids was calculated per mg equivalents of quercetin per gram of each extract.

### 2.4. Determination of Total Flavonols

The flavonols content was measured according to the previously published method [6]. The dill extract (1 mg/mL) was added to 2 mL of AlCl_3_ (20 mg/mL) and 6 mL sodium acetate solution (50 mg/mL). The mixture was then incubated for 150 min at room temperature and the absorbance was determined at 440 nm. The content of total flavonols was calculated per mg equivalents of quercetin per gram of each extract.

### 2.5. Experimental Design

A total of 36 male golden Syrian hamsters weighing 130 ± 10 g were used in this experiment. Animals were kept for one week before experimentation and adaptation. Regular conditions with light/dark cycle (12 hour for each), relative humidity of 60 ± 5%, and temperature at 23 ± 2°C were applied during the experiments. After adaptation, hamsters were randomly divided into six experimental groups (*n* = 6) and fed as follows: group 1: chow + 2% cholesterol + 0.5% cholic acid; group 2: chow + 100 mg/kg hydroalcoholic extract of dill + 2% cholesterol + 0.5% cholic acid; group 3: chow + 200 mg/kg hydroalcoholic extract of dill + 2% cholesterol + 0.5% cholic acid; group 4: chow + 100 mg/kg dill tablet + 2% cholesterol + 0.5% cholic acid; group 5: chow + 200 mg/kg dill tablet + 2% cholesterol + 0.5% cholic acid; and group 6: chow. At the end of 30 days' experiment, animals were anesthetized and sacrificed after 12 h of fasting. Blood samples were collected from the heart and serum was separated by centrifugation of samples at 3000 rpm for 10 min.

### 2.6. Determination of Serum Factors

Liver enzymes, total cholesterol, fasting blood sugar, triglyceride, and HDL-C were measured by colorimetric methods using the commercial available kits (Pars Azmun, Tehran, Iran), whereas LDL-C and VLDL-C were calculated by Friedwald's formula [8, 9].

### 2.7. RNA Extraction and cDNA Synthesis

Total RNA was isolated from liver tissue samples (100 mg) using TRIZOL reagent according to the manufacturer's protocol (Fermentas Life Sciences, Vilnius, Lithuania). The integrity of extracted RNA was determined by 1% agarose gel while its concentration and purity were assessed by Nano-Drop spectrophotometer (Bio-TeK, USA). After that, 1 microgram of RNA was applied for reverse transcription process using QuantiTect Reverse Transcription Kit (Fermentas Life Sciences, Vilnius, Lithuania). Integrity, purity, and concentration of cDNA were also measured as described for RNA [8].

### 2.8. Real-Time qRT-PCR Assay

Determination of HMG-CoA reductase mRNA level was carried out by real-time PCR using QuantiTect SYBR Green PCR Kit (Qiagen Ltd., Hilden, Germany) in a PCR detection machine (Bio Rad Ltd., USA).

Primers of reaction were designed by AlleleID7 software (Premier Biosoft Corporation, USA) for HMG-CoA (forward: 5′-AAGGAGCGTGCAAAGACAATC-3′ and reverse: 5′-TGAACCATGTGACTTCTAACAAG-3′) and GAPDH (forward: 5′-TGGCCTTCCTTCCTACG-3′ and reverse: 5′-TAGCCCAGGATGCCCTTCAG-3′) as internal control. The RT-PCR reaction mixture was prepared by the addition of 1 *μ*L cDNA template (<100 ng), 10 *μ*L SYBR Premix (1X), and 1 *μ*L of each primer (0.4 *μ*M) and adjusting of volume to 20 *μ*L by adding deionized water. HMG-CoA reductase mRNA level was measured in triplicate in 38 cycles using thermal cycler.

### 2.9. HMG-CoA Reductase Activity

Liver tissue samples were homogenized in 500 *μ*L lysis buffer containing 1% protein inhibitor (Sigma-Aldrich Co., St. Louis, MO, USA) and centrifuged for 15 minutes at 14000 rpm and 4°C. Supernatant was used for the measurement of enzyme activity using HMG-CoA reductase assay kit (Sigma-Aldrich Co., St. Louis, MO, USA) based on spectrophotometrical determination of oxidized NADPH at 340 nm.

### 2.10. Statistical Analysis

Data are expressed as means ± SEM of three replicate measurements and then analyzed by SPSS package (version 16, SPSS, Inc.). One-way analysis of variance (ANOVA) followed by Dunnett's test were used. The *p* values less than 0.05 were regarded statistically significant.

## 3. Results

Total phenol, flavonoid, and flavonol contents of dill tablet and dill extract are shown in Table 1.

**Figure 1:**
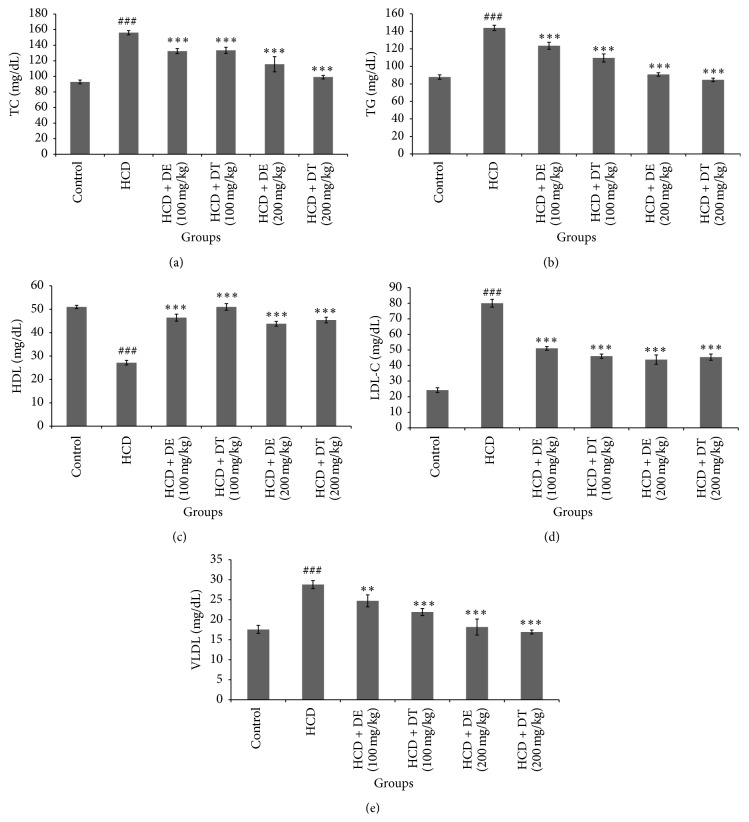
Serum total cholesterol, TG, LDL-C, HDL-C, and VLDL-C levels in different groups of hamsters after one month of treatment with dill tablet or extract. ^*∗∗∗*^ *p* < 0.001 and ^*∗∗*^ *p* < 0.01 compared with high cholesterol diet. ^###^ *p* < 0.001 compared with control. Data are presented as means ± SEM.

**Figure 2:**
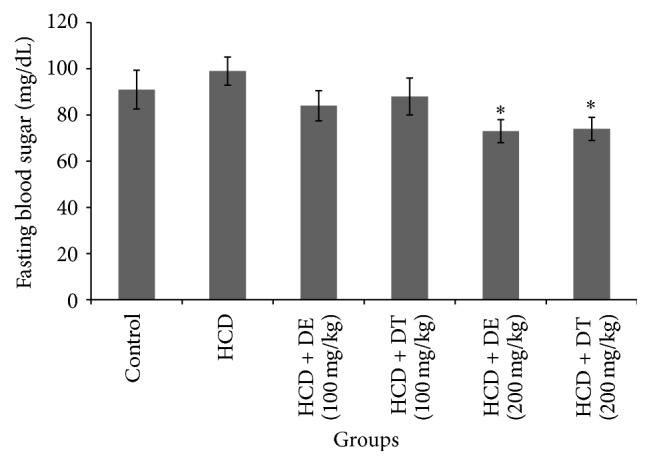
Fasting blood glucose levels in different groups of hamsters after one month of treatment with dill tablet or extract. ^*∗*^ *p* < 0.05 compared with high cholesterol diet.

**Figure 3:**
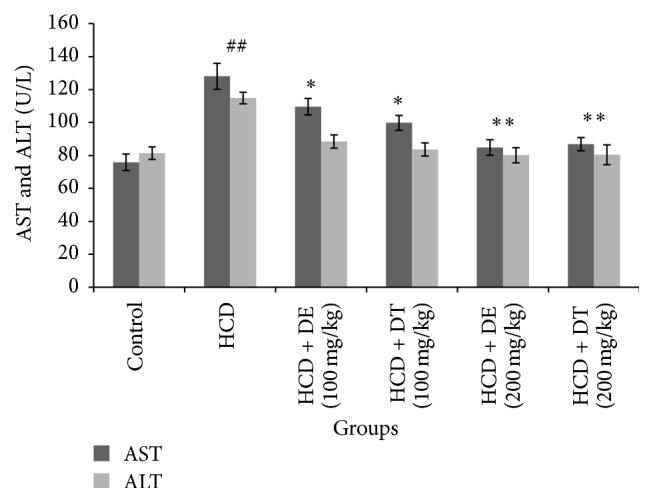
Serum ALT and AST levels in different groups of hamsters after four weeks of treatment with dill tablet and extract. ^*∗∗*^ *p* < 0.01 and ^*∗*^ *p* < 0.05 compared with high cholesterol diet. ^##^ *p* < 0.001 compared with control. Data are presented as means ± SEM.

Figure 1 shows mean of FBS (fasting blood sugar), total cholesterol, triglyceride, LDL-C, HDL-C, and VLDL-C levels in the experimental groups. Serum levels of total cholesterol, triglyceride, LDL-C, and VLDL-C significantly reduced (*p* < 0.05) in dill tablet and dill extract treated hamsters. These reductions were higher in hamsters treated with 200 mg/kg of dill extract or dill tablet. Significant increase was observed in HDL-C level by treatment with 100 and 200 mg/kg of dill extract or dill tablet. Treatment of hamsters with 100 mg/kg of dill extract or dill tablet did not affect body weight compared with untreated high cholesterol diet fed hamsters (159 ± 6 or 155 ± 5.8 g versus 168 ± 5.1 g). However, there was a marked weight gain in high cholesterol diet fed animals compared with control (166 ± 5.5 versus 140 ± 4.0 g). Receiving 200 mg/kg of dill extract or dill tablet significantly (*p* < 0.05) reduced the body weight compared to the animals receiving only high cholesterol diet (146 ± 3.9 or 148.1 ± 4.4 g versus 168 ± 5.1 g). Fasting blood glucose levels significantly declined in animals treated with 200 mg/kg of dill extract or dill tablet as indicated in Figure 2. Dill extract and dill tablet at the dose of 100 and 200 mg/kg significantly normalized liver enzymes (ALT and AST) in hypercholesterolemic group (Figure 3).

The effect of dill tablet and dill extract on gene expression of HMG-CoA was determined using real-time PCR technique. To show the relative changes in the expression of this gene between the studied groups, ΔCt and ΔΔCt were calculated; subsequently fold change of expression was calculated using 2^−ΔΔCt^ method. The results are summarized in Table 2. These analyses showed that high cholesterol diet reduced HMG-CoA reductase gene expression compared to normal group.

Furthermore, according to statistical analysis of ΔΔCt, dill tablet and dill extract at 200 mg/kg dose reduced HMG-CoA reductase in treated group comparing to HCD group (*p* < 0.001).

**Figure 4:**
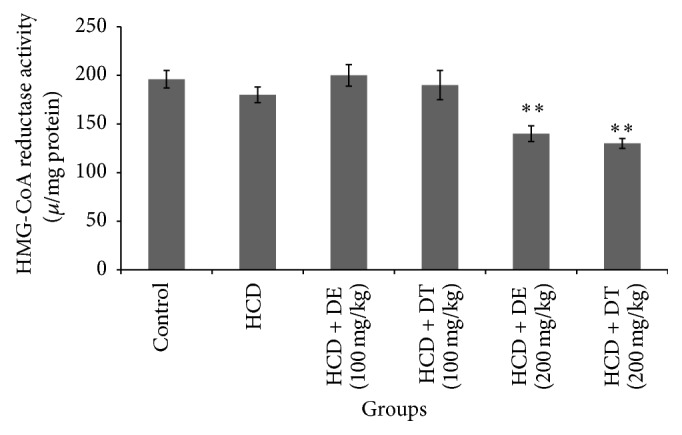
HMG-CoA reductase activity in different groups of hamsters after four weeks of treatment with dill tablet and extract. ^*∗∗*^ *p* < 0.01 compared with high cholesterol diet and control. Data are presented as means ± SEM.

In addition, dill extract or dill tablet-receiving hamsters (200 mg/kg) showed significantly reduced HMG-CoA reductase activity compared with high cholesterol diet fed or control hamsters (*p* < 0.05), while the changes made in enzyme activity after treatment of animals with 100 mg/kg of dill extract or dill tablet did not reach statistical significance (Figure 4).

## 4. Discussion

Many epidemiological studies have shown a direct relationship between lipid abnormalities, particularly increased levels of total cholesterol and LDL-C, and the risk of coronary vascular diseases (CVD) [1]. Suppression of HMG-CoA reductase activity, the rate-limiting enzyme in the of cholesterol biosynthesis pathway, causes significant reduction in VLDL-C production by the liver, upregulation of liver LDL receptor, and consequently clearance of LDL-C from blood circulation [10]. In this study, total cholesterol, triglycerides, LDL-C, and VLDL-C significantly reduced, while HDL-C increased in dill extract and dill tablet treated animals. Our results are in agreement with the previous studies by Yazdanparast and Bahramikia [11], Setorkil et al. [12], and Yazdanpanah [7] who confirmed that dill significantly declines blood glucose, cholesterol, triglyceride, LDL-C, and VLDL-C and also increases HDL-C level in the diabetic, hyperlipidemic, and metabolic syndrome patients as well as in experimental animal models.

This study presents preliminary results indicating that dill tablet and dill extract are able to decline total cholesterol levels by inhibiting liver HMG-CoA reductase activity. The main approach involved in hypercholesterolemia treatment is the suppression of cholesterol synthesis by declining of HMG-CoA reductase activity which is known as the rate-limiting enzyme in the cholesterol biosynthesis pathway [13]. HMG-CoA reductase gene expression was significantly reduced in high cholesterol diet fed groups that were treated by 100 and 200 mg/kg of dill tablet and dill extract.

However, only dill tablet and dill extract at the dose of 200 mg/kg reduced HMG-CoA reductase activity. As we expected, HMG-CoA reductase activity reduced in high cholesterol diet fed group. According to these findings, we suggest that 200 mg/kg dose of dill possibly exerts its regulatory effect at the transcriptional, translational, and posttranslational levels.

But dill at the dose of 100 mg/kg only regulated at the transcriptional levels, and enzyme activity was not affected at this dose. Further experiments are required to determine the exact mechanism in HMG-CoA reductase activity inhibition.

No exact mechanism has been recognized for hypocholesterolemic effects of dill so far. Yazdanparast and Bahramikia [11] has shown that HMG-CoA/mevalonate ratio is altered after treatment of rats with dill extract. Haghighi et al. [14] examined the activity of liver phosphatidate phosphohydrolase enzyme as possible pathway for the lipid lowering effect of dill. They postulated that phenolic components are responsible for the hypocholesterolemic activity of dill possibly via the changes in HMG-CoA reductase or acyl-CoA carboxylase activities. For the first time we showed that dill normalized lipid profile by decreasing HMG-CoA reductase activity in the hamsters. Furthermore dill tablet treated animals showed more reduction in the HMG-CoA reductase activity compared with dill extract treated groups. In another study, we also showed that dill increased LDL receptor in the liver, resulting in stimulation of cholesterol clearance from blood stream (unpublished data).

Some studies have reported that rutin and quercetin (components of dill) decreased serum total cholesterol and LDL-C and reduced liver cholesterol levels [15]. Other studies have also reported that quercetin reduced HMG-CoA reductase activity [16]. Therefore, it is likely that the quercetin content of dill may be responsible for the inhibition of HMG-CoA reductase activity.

Effects of the antioxidant agents (including flavonoids, phenolics, and tannins) on the control of endogenous cholesterol biosynthesis have previously been investigated and antiatherosclerosis properties of these compounds have been reported in different experiments [17]. For example, Min and Kim [18] reported that kakkalide and irisolidone (two types of flavonoids) significantly reduce HMG-CoA reductase activity. Lee et al. [19] also showed that naringenin (a type of flavonoids) suppresses HMG-CoA reductase activity and significantly reduces plasma and hepatic lipids in high cholesterol fed animals. Chen et al. [20] and Kwon et al. [21] established that flavonoids isolated from different herbal medicine significantly inhibit HMG-CoA reductase activity.

Our HPLC analysis showed that AG (leaves and seeds) contains different type of flavonoids and monoterpenoid including limonene, linalool, quercetin, *α*-pinene, phenol, flavonoid, flavonol, anthocyanin, tannins, saponin, and alkaloid [22]. Salvamani et al. [23] reported that different types of plant flavonoids have potential antiartherosclerotic effects. Peffley and Gayen [24] reported that plant-derived monoterpenes inhibit HMG-CoA reductase synthesis at the posttranscriptional level.

Phenolic compounds also have antioxidant and scavenging activity as well as involvement in the inhibition of lipid peroxidation, mutagenesis, carcinogenesis, atherogenesis, and thrombosis [17]. In the present study, moderate amounts of phenolics, flavonoids, and flavonols were detected in dill tablet and dill extract. These components probably are responsible for the useful effects of dill. Interestingly, we recently reported that different extract of dill has potential antioxidant activity [6, 25].

An increased level of serum ALT and AST was observed in hypercholesterolemic group, while these enzymes significantly reduced in dill tablet and dill extract treated animals. Serum AST and ALT have been recognized as sensitive marker of liver injury. In fact, damage of hepatocytes changes membrane permeability and cell function and causes the leakage of these enzymes into the blood stream [26]. High cholesterol diet gives a rise in oxidative stress in the liver, causes an increase in liver enzymes, and leads to formation of fatty liver [27].

In this study, blood glucose significantly reduced after treatment by dill tablet or dill extract (200 mg/kg). This observation is in line with previous studies by Mishra [28] and Madani et al. [29] confirming that aqueous extract of dill markedly reduced glucose levels in diabetic animals. This observation has further been confirmed by Mobasseri et al. [30] which showed that administration of dill in diabetic patients normalized insulin sensitivity and reduced lipid profiles.

Since we did not measure the consumed food for each animal, it can be mentioned as a limitation of this study. Furthermore, since alteration in mRNA level does not necessarily reflect changes in the related protein, determination the produced protein can indicate more precise mechanisms.

Study of cellular mechanisms also can help in better interpretation of the results.

## 5. Conclusions 

This study established that dill has potential hypolipidemic activity; consequently it can be used in hypercholesterolemic patients. On the other hand, this study revealed the cholesterol lowering effect of dill via inhibition of HMG-CoA reductase activity in hamsters. Further experiments are also needed to determine the similar properties of dill in hypercholesterolemic patients.

## Figures and Tables

**Table 1:** Total phenols, flavonoids, and flavonols content of dill tablet and dill extract.

Photochemical components	Dill tablet	Dill extract
Total phenols (mg equivalent of gallic acid/g)	190 ± 4.4	160 ± 4.0
Total flavonoids (mg equivalent of quercetin/g)	151 ± 3.5	120 ± 5.3
Total flavonols (mg equivalent of quercetin/g)	135 ± 3.0	101 ± 4.0

**Table 2:** The effects of dill tablet (DT) and dill extract (DE) on HMG-CoA reductase gene expression (fold change).

Groups	Fold change (2^−ΔΔCt^)
HCD/normal	2.50
DT 100/HCD	0.70
DT 200/HCD	0.27
DE 100/HCD	0.40
DE 200/HCD	0.47

HCD: high cholesterol diet, DT 100 and DT 200: HCD group treated with 100 and 200 mg/kg dill tablet, respectively; DE 100 and DE 200: HCD group treated with 100 and 200 mg/kg dill extract, respectively (*n* = 6 in each group).

## References

[1] Stone G. W., Maehara A., Lansky A. J. (2011). A prospective natural-history study of coronary atherosclerosis. *The New England Journal of Medicine*.

[2] Steinberg D. (2006). An interpretive history of the cholesterol controversy. Part V. The discovery of the statins and the end of the controversy. *Journal of Lipid Research*.

[3] Pahan K. (2006). Lipid-lowering drugs. *Cellular and Molecular Life Sciences*.

[4] Jick H., Zornberg G. L., Jick S. S., Seshadri S., Drachman D. A. (2000). Statins and the risk of dementia. *The Lancet*.

[5] Mohammadi A., Mirzaei F., Jamshidi M. (2013). Influence of flaxseed on lipid profiles and expression of LXRa, in intestine of diabetic rat. *International Journal of Biology*.

[6] Oshaghi E. A., Tavilani H., Khodadadi I., Goodarzi M. T. (2015). Dill tablet: a potential antioxidant and anti-diabetic medicine. *Asian Pacific Journal of Tropical Biomedicine*.

[7] Yazdanpanah K. (2001). Effects of dill juice on serum low density lipoprotein, triglyceride and high density lipoprotein in patients with hyperlipidemia. *Scientific Journal of Kurdistan University of Medical Sciences*.

[8] Mohammadi A., Vafaei S. A., Nabi Moradi M., Ahmadi M., Pourjafar M., Abbasi Oshaghi E. (2015). Combination of ezetimibe and garlic reduces serum lipids and intestinal niemann-pick C1-like 1 expression more effectively in hypercholesterolemic mice. *Avicenna Journal of Medical Biochemistry*.

[9] Mohammadi A., Mirzaei F., Moradi M. (2013). Effect of flaxseed on serum lipid profile and expression of NPC1L1, ABCG5 and ABCG8 genes in the intestine of diabetic rat. *Avicenna Journal of Medical Biochemistry*.

[10] Goldstein J. L., Brown M. S. (2009). The LDL receptor. *Arteriosclerosis, Thrombosis, and Vascular Biology*.

[11] Yazdanparast R., Bahramikia S. (2008). Evaluation of the effect of *Anethum graveolens* L. crude extracts on serum lipids and lipoproteins profiles in hypercholesterolaemic rats. *DARU*.

[12] Setorkil M., Shahinfard N., Ansari R., Foronzandeh Z., Asgharzadeh S., Rafieian-Kopaei M. (2013). Comparison between the effects of hydroalcoholic extract of Dill and statins on lipid profile. *Journal of Kerman University of Medical Sciences*.

[13] Xu G., Salen G., Shefer S. (1996). Increasing hepatic cholesterol 7*α*-hydroxylase reduces plasma cholesterol concentrations in normocholesterolemic and hypercholesterolemic rabbits. *Hepatology*.

[14] Haghighi B., Kharazizadeh M., Attar M. A. (2007). Possible involvement of hepatic phosphatidate phosphohydrolase in the mechanisms of actions of certain antilipemic drugs in rats. *Iranian Journal of Pharmaceutical Research*.

[15] Arai Y., Watanabe S., Kimira M., Shimoi K., Mochizuki R., Kinae N. (2000). Dietary intakes of flavonols, flavones and isoflavones by Japanese women and the inverse correlation between quercetin intake and plasma LDL cholesterol concentration. *The Journal of Nutrition*.

[16] Sung J. H., Lee S.-J., Park K. H., Moon T. W. (2004). Isoflavones inhibit 3-hydroxy-3-methylglutaryl coenzyme A reductase in vitro. *Bioscience, Biotechnology and Biochemistry*.

[17] Knekt P., Kumpulainen J., Järvinen R. (2002). Flavonoid intake and risk of chronic diseases. *The American Journal of Clinical Nutrition*.

[18] Min S.-W., Kim D.-H. (2007). Kakkalide and irisolidone: HMG-CoA reductase inhibitors isolated from the flower of *Pueraria thunbergiana*. *Biological and Pharmaceutical Bulletin*.

[19] Lee M.-K., Moon S.-S., Lee S.-E. (2003). Naringenin 7-*O*-cetyl ether as inhibitor of HMG-CoA reductase and modulator of plasma and hepatic lipids in high cholesterol-fed rats. *Bioorganic & Medicinal Chemistry*.

[20] Chen T.-H., Liu J.-C., Chang J.-J., Tsai M.-F., Hsieh M.-H., Chan P. (2001). The in vitro inhibitory effect of flavonoid astilbin on 3-hydroxy-3-methylglutaryl coenzyme a reductase on vero cells. *Zhonghua Yi Xue Za Zhi*.

[21] Kwon E. K., Lee D. Y., Lee H. (2010). Flavonoids from the buds of *Rosa damascena* inhibit the activity of 3-hydroxy-3-methylglutaryl-coenzyme a reductase and angiotensin I-converting enzyme. *Journal of Agricultural and Food Chemistry*.

[22] Abbasi Oshaghi E. (2015). *Study of biochemical cheracteristic, metabolic effects and some mechanism of dill tablet in type 2 diabetic rat [Ph.D. thesis]*.

[23] Salvamani S., Gunasekaran B., Shaharuddin N. A., Ahmad S. A., Shukor M. Y. (2014). Antiartherosclerotic effects of plant flavonoids. *BioMed Research International*.

[24] Peffley D. M., Gayen A. K. (2003). Plant-derived monoterpenes suppress hamster kidney cell 3-hydroxy-3-methylglutaryl coenzyme a reductase synthesis at the post-transcriptional level. *Journal of Nutrition*.

[25] Abbasi Oshaghi E., Khodadadi I., Tavilani H., Goodarzi M. T. Aqueous extract of *Anethum graveolens* L. (dill) has potential antioxidant and antiglycation effects. *Iranian Journal of Medical Sciences*.

[26] Nyblom H., Berggren U., Balldin J., Olsson R. (2004). High AST/ALT ratio may indicate advanced alcoholic liver disease rather than heavy drinking. *Alcohol and Alcoholism*.

[27] Bugianesi E., Gentilcore E., Manini R. (2005). A randomized controlled trial of metformin versus vitamin E or prescriptive diet in nonalcoholic fatty liver disease. *The American Journal of Gastroenterology*.

[28] Mishra N. (2013). Haematological and hypoglycemic potential Anethum graveolens seeds extract in normal and diabetic Swiss albino mice. *Veterinary World*.

[29] Madani H., Ahmady Mahmoodabady N., Vahdati A. (2005). Effects of hydroalchoholic extract of Anethum graveolens (Dill) on plasma glucose an lipid levels in diabetes induced rats. *Iranian Journal of Diabetes and Metabolism*.

[30] Mobasseri M., Payahoo L., Ostadrahimi A., Bishak Y. K., Jafarabadi M. A., Mahluji S. (2014). Anethum graveolens supplementation improves insulin sensitivity and lipid abnormality in type 2 diabetic patients. *Pharmaceutical Sciences*.

